# Supplementary material for: Inoculation with Actinobacteria spp. Isolated from a Hyper-Arid Environment Enhances Tolerance to Salinity in Lettuce Plants (Lactuca sativa L.)
Source: Plants (Basel). 2023 May 18;12(10):2018. doi: 10.3390/plants12102018 (PMC10222102; doi:10.3390/plants12102018)
Supplement: Supplementary file 1 [file plants-12-02018-s001.zip › plants-2392870-supplementary.pdf]

Table S1. Absorption of Mg^{+}, Ca^{+}, P^{-} in the root and shoot.

Inoculo	Mg^{+} Root (mg g^{-1})	Mg^{+} Leaves (mg g^{-1})	Ca^{+} Root (mg g^{-1})	Ca^{+} Leaves (mg g^{-1})	P^{-} Root (mg g^{-1})	P^{-} Leaves (mg g^{-1})
**0 mM NaCl**						
Control	6,04 ± 0,43 bc	6,86 ± 0,51 bc	3,43 ± 0,24 c	10,4 ± 1,87 d	1,69 ± 0,06 a	2,37 ± 0,17 ab
PSC3	7,22 ± 0,13 ab	7,7 ± 0,92 abc	4,54 ± 0,02 abc	16,6 ± 4,13 abc	1,46 ± 0,12 ab	2,57 ± 0,2 a
SAC22	5,92 ± 0,79 c	9,13 ± 0,86 a	3,91 ± 0,43 bc	20,7 ± 1,53 a	1,49 ± 0,03 ab	2,22 ± 0,05 ab
SNC13	7,19 ± 0,47 ab	8,65 ± 0,03 ab	4,99 ± 0,46 ab	19,2 ± 1,44 ab	1,39 ± 0,04 abc	1,70 ± 0,11 cd
SLC12	7,47 ± 0,08 a	6,41 ± 1,16 c	5,04 ± 0,04 ab	10,8 ± 2,32 d	1,28 ± 0,25 bc	2,23 ± 0,2 ab
**100 mM NaCl**						
Control	3,11 ± 0,22 d	7,2 ± 0,15 bc	4,03 ± 0,31 abc	14,92 ± 0,23 bcd	1,53 ± 0,04 ab	1,70 ± 0,04 cd
PSC3	3,51 ± 0,11 d	4,09 ± 0,57 d	4,19 ± 0,49 abc	11,45 ± 1,46 cd	1,43 ± 0,09 abc	2,09 ± 0,14 abc
SAC22	3,61 ± 0,12 d	6,01 ± 0,26 c	5,14 ± 0,21 a	16,02 ± 1,35 abcd	1,54 ± 0,04 ab	1,98 ± 0,16 bcd
SNC13	2,6 ± 0,83 d	6,43 ± 0,67 c	4,12 ± 0,86 abc	14,38 ± 1,93 bcd	1,5 ± 0,15 ab	1,49 ± 0,35 d
SLC12	3,75 ± 0,12 d	7,27 ± 0,21 bc	5,06 ± 0,22 ab	19,33 ± 0,39 ab	1,12 ± 0,02 c	2,17 ± 0,15 abc

Data represent the mean values of three replicates ± SD. Different letters indicate a significant difference in Auxin production according to LSD fisher test at $p < 0.05$.

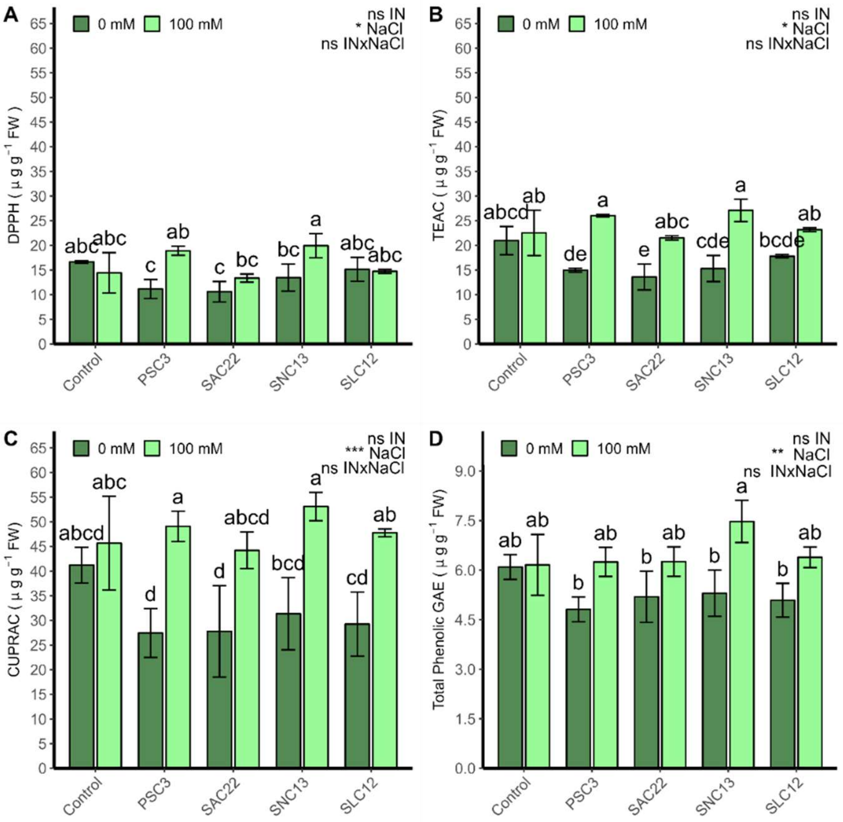

Figure S1. Phenolic compounds and antioxidant activities of leaves of lettuce plants noninoculated (Control), inoculated by *P. siccitolerans* ATMLC32022 (PSC3), *S. ambofaciens* ATMLC222021 (SAC22), *S. niveoruber* ATMLC132021 (SNC13) and *S. lienomycini* ATMLC122021 (SLC12) under salinity stress (100 mM of NaCl light green) and in absence of salt (0 mM of NaCl dark green). (A) Antioxidant activity (AA) determined by DPPH, (B) TEAC, (C) CUPRAC method and (C) total phenol concentrations. The data includes means ± SE (n = 4). The data was analyzed through a two-way ANOVA conducted with inoculation and salinity stress as sources of variation, the significant difference was depicted as, ns: non-significant, $P<0.05$: * and $P<0.0001$: ***. Different letters indicate significant differences ($P \leq 0.05$) according to Fisher's multiple range test.

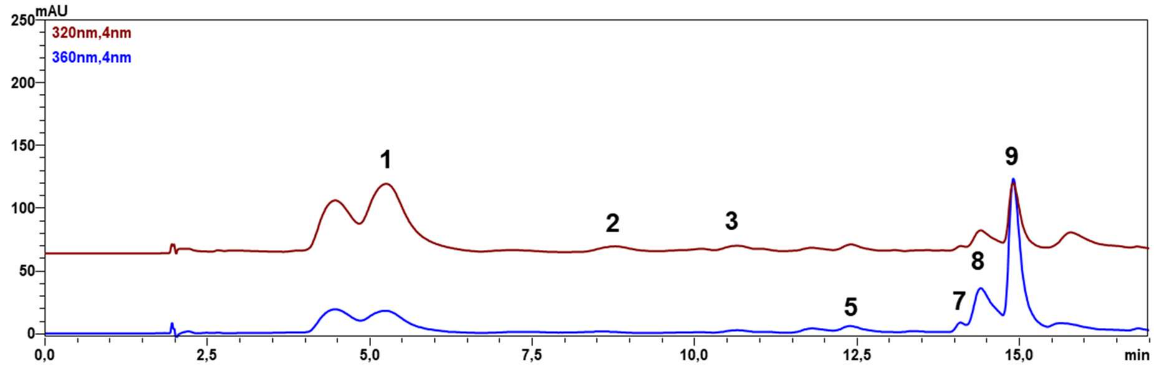

Figure S2**.** HPLC-DAD chromatogram (320 and 360 nm) of phenolic compounds as flavonols and phenolic acids in lettuce leaves growing under saline conditions.

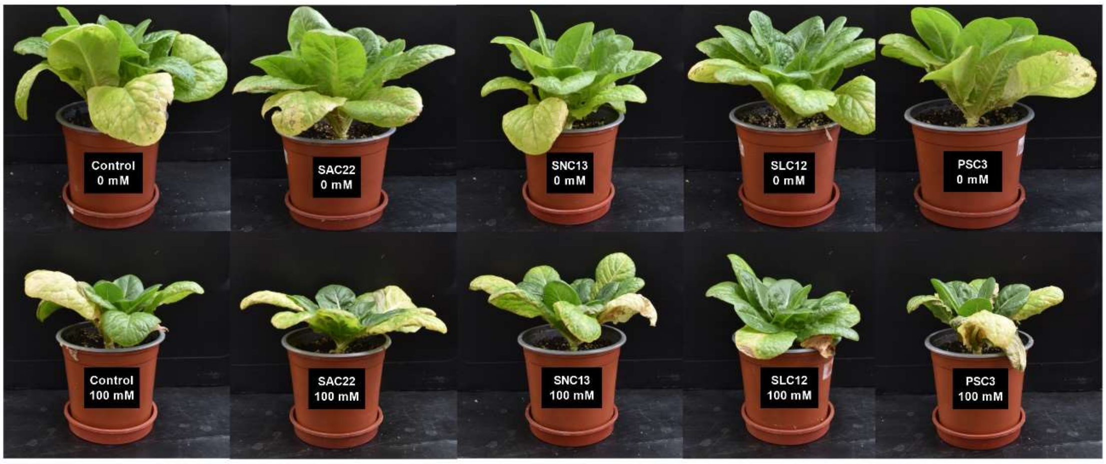

Figure S3**.** Phenotypic comparison of lettuce plants (*Lactuca sativa* L) noninoculated (Control), inoculated by *P. siccitolerans* ATMLC32022 (PSC3), *S. ambofaciens* ATMLC222021 (SAC22), *S. niveoruber* ATMLC132021 (SNC13) and *S. lienomycini* ATMLC122021 (SLC12) under salinity stress (100 mM of NaCl) and in absence of salt (0 mM of NaCl).
